# Kyrieleis Arteriolitis Associated with Acute Retinal Necrosis due to Herpes Simplex Virus Type 1 Secondary to Herpetic Encephalitis

**DOI:** 10.3390/vision6020027

**Published:** 2022-05-18

**Authors:** Olga E. Makri, Iasonas K. Tsekouras, Leonidia Leonidou, Konstantinos Kagkelaris, Vassilios Kozobolis, Constantinos D. Georgakopoulos

**Affiliations:** 1 Department of Ophthalmology, Medical School, University of Patras, 265 04 Patras, Greece; makriolga@upatras.gr (O.E.M.); iktsek@yahoo.gr (I.K.T.); kostas.kagkelaris@gmail.com (K.K.); kozobolis@gmail.com (V.K.); 2 Internal Medicine, Department of Internal Medicine, Patras University Hospital, 265 04 Patras, Greece; lydleon@yahoo.gr

**Keywords:** acute retinal necrosis, herpes simplex, Kyrieleis, foscarnet

## Abstract

We report the case of a 52-year-old woman who presented to the emergency department with acute retinal necrosis in her left eye secondary to herpes simplex virus type 1 encephalitis for which she had been hospitalized four months before. Treatment with intravitreal foscarnet and intravenous acyclovir was promptly commenced followed by the addition of oral prednisolone. PCR analysis of aqueous humor detected HSV type 1 DNA. The condition responded to therapy with partial resolution of intraocular inflammation and improvement of visual acuity, but the presence of Kyrieleis plaques was observed two weeks after the initiation of treatment, when five intravitreal foscarnet injections had been administered. The patient was switched to oral therapy with valacyclovir, and 10 weeks after commencing treatment, the patient’s left eye was free of inflammation, having achieved a BCVA of 20/20. Oral steroid treatment was gradually tapered off, and the patient was instructed to remain on prophylactic antiviral therapy. Kyrieleis arteriolitis is an uncommon finding in the context of acute retinal necrosis. As far as we are aware, we report the first case of Kyrieleis arteriolitis in acute retinal necrosis secondary to viral encephalitis and the second one presenting Kyrieleis plaques in acute retinal necrosis caused by herpes simplex virus type 1. Prior reports of cases of Kyrieleis arteriolitis in acute retinal necrosis are also presented.

## 1. Introduction

Acute retinal necrosis (ARN) is a severe inflammatory condition characterized by panuveitis and occlusive retinal vasculitis, mainly with arteriolar involvement, with subsequent peripheral retinal necrosis. Circumferential spread of retinal necrosis, which is rapid in the absence of treatment, is among the diagnostic criteria for the disease [1,2]. Acute retinal necrosis is usually attributed to varicella zoster virus (VZV) and herpes simplex virus (HSV) (type 1 and 2) infection. It is rarely considered as a clinical manifestation of cytomegalovirus or Epstein-Barr virus infection. Immunocompetent patients seem to be preferentially affected. Herpes simplex virus tends to be responsible for ARN in younger patients and VZV is usually the cause of this viral syndrome in older patients [3]. The diagnosis of ARN is usually made on clinical grounds, and polymerase chain reaction, performed on intraocular fluids, is used for the detection of viral DNA so that the responsible virus of the herpes family can be determined. The effect of ARN on visual function is frequently devastating due to severe inflammation and ischemia of the retina and optic nerve and the resulting retinal and optic atrophy. The possibility of rhegmatogenous retinal detachment in patients with ARN also contributes to the poor prognosis of the visual outcome [4].

## 2. Case Report

**Figure 1:**
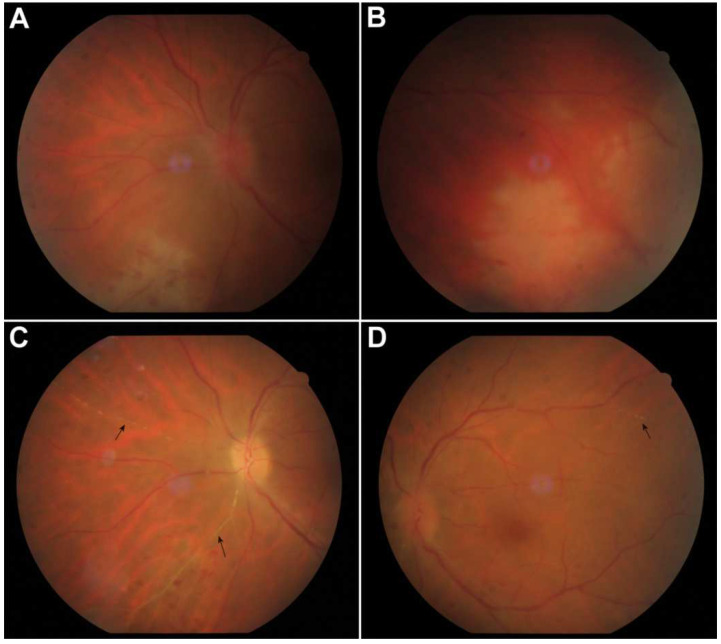
Color fundus photo of the left eye at presentation showing vitreous inflammation, optic nerve edema and peripheral areas of retinal necrosis with neighboring vasculitis in the temporal, inferior and nasal quadrants (**A**,**B**). Two weeks after the initiation of treatment, vitritis decreased and Kyrieleis plaques were seen in nasal and temporal retinal arteries, as yellowish plaques that did not extend beyond the vessel walls ((**C**,**D**), arrows).

A 52-year-old Caucasian female presented to the emergency department with a 3-day history of blurred vision, redness, floaters and photophobia in her left eye (OS). Her previous ocular history was unremarkable, but she reported that she had been hospitalized 4 months before due to encephalitis, the causative agent of which had been shown to be HSV type 1. During the ophthalmological examination, the patient’s best corrected visual acuity (BCVA) was 20/20 in the right eye (OD) and 20/33 in OS. The anterior segment examination revealed dilated ciliary blood vessels and intense anterior chamber activity in OS. The dilated fundus examination in OS showed prominent vitritis, coalescing areas of peripheral retinal necrosis from2 o’clock over 6 o’clock to 10 o’clock without involvement of the posterior pole and mild optic disk edema (Figure 1A,B). The slit-lamp examination and fundoscopy in OD were unremarkable. Patient’s clinical data are summarized in Table 1.

As the aforementioned clinical signs were typical of ARN, the patient was admitted to our hospital where she promptly received an intravitreal injection of foscarnet at a dose of 2.4 mg/0.1 mL. An aqueous humor sample was obtained and sent for polymerase chain reaction analysis prior to the intravitreal administration of foscarnet. Treatment with intravenous acyclovir 13 mg/kg/day in three divided doses was started, and oral prednisolone 1 mg/kg/day was added 36 h later. Polymerase chain reaction analysis of the collected aqueous humor detected HSV type 1 DNA.

**Figure 2:**
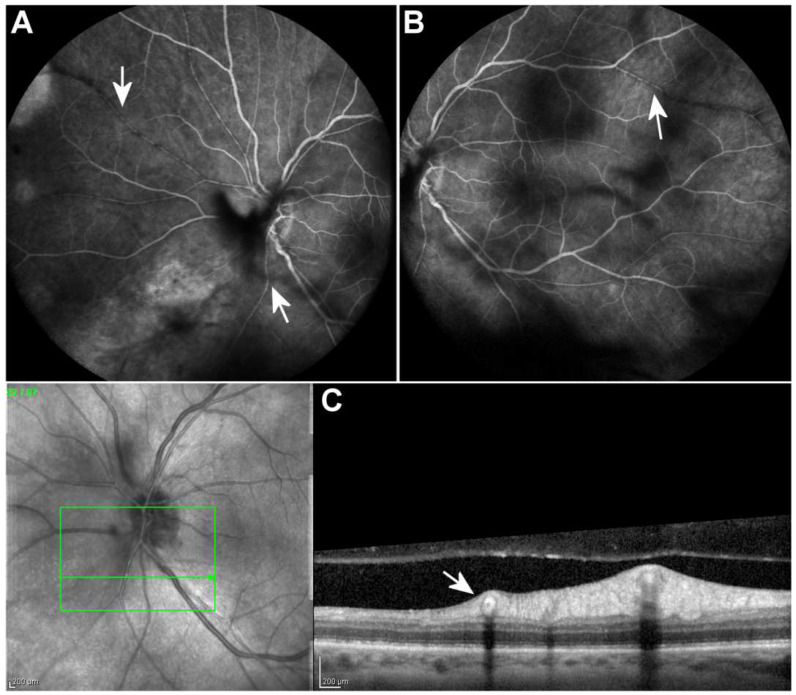
Fluorescein angiography 2 weeks after the initiation of treatment revealed normal arterial filling and absence of dye leakage from the Kyrieleis plaques ((**A**,**B**), arrows). Optical coherence tomography scan along the affected vessel shows hyperreflectivity of the entire wall of the vessels ((**C**), arrow). Scale bar: 200 µm.

Two weeks after commencing therapy, five intravitreal injections of foscarnet had been administered. At that point, OD remained unaffected, and the examination of OS revealed partial resolution of inflammatory activity in the anterior chamber and vitreous as well as active retinal inflammation. The patient’s BCVA in OS improved to 20/26. However, during the dilated fundus examination and fluorescein angiography, multiple Kyrieleis plaques along several arterioles were observed (Figure 1C,D and Figure 2A,B, arrows).

**Figure 3:**
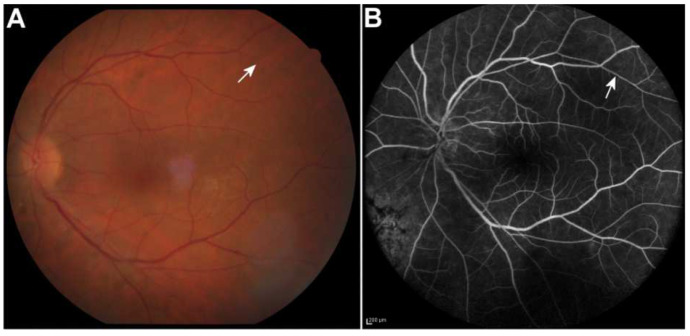
The Kyrieleis plaques faded with a few persisting at 3 months after the initiation of treatment in color fundus photo (**A**) and fluorescein angiography (**B**) of the left eye (arrows). Scale bar 200 µm.

In the following 6 weeks, the patient was on oral valacyclovir 3 g/day in three divided doses, tapering of prednisolone was started and the inflammation continued subsiding with complete resolution 10 weeks after commencing treatment, at which point the patient’s BCVA in OS was 20/20. Due to the resolution of vitritis, a clearer view of the fundus could be obtained, and the presence of Kyrieleis arteriolitis became evident. Retinal atrophy in the area of peripheral lesions was observed. Oral prednisolone was gradually tapered off, and the patient remained on prophylactic oral valacyclovir therapy (1 g/day). The Kyrieleis plaques subsequently faded with a few persisting at 3 months (Figure 3).

## 3. Discussion

We described a case of ARN associated with Kyrieleis arteriolitis HSV type 1 encephalitis. Several cases of ARN after viral encephalitis have been reported, and HSV type 1 has been recognized as the causative agent in some of them. Herpes simplex encephalitis has been reported to be the most common cause of viral encephalitis, with HSV type 1 being responsible for approximately 90% of the former [5,6]. As herpetic encephalitis appears to be a risk factor for ARN, the possibility of such an insult may serve as an additional reason for prophylactic antiviral treatment after herpetic encephalitis to be considered [7].

The case we described depicts a particularly uncommon presentation of ARN with Kyrieleis arteriolitis. Kyrieleis plaques seem to consist of whitish glistening segmental deposits of immune cells and inflammatory debris scattered along arterioles within or adjacent to their walls. However, their pathogenesis remains unknown due to the lack of relevant pathological studies. Pichi et al. conducted a multimodal imaging study of 25 eyes with Kyrieleis arteritis and suggested that Kyrieleis plaques represent an inflammatory process within the vessel wall and most probably the endothelium. Full-thickness involvement of the vessel wall is highly unlikely, as indicated by the absence of leakage in fluorescein angiography [8,9]. Kyrieleis arteriolitis has primarily been associated with ocular toxoplasmosis, but it has also been described in tuberculosis, syphilis, acute retinal necrosis, cytomegalovirus retinitis and Mediterranean spotted fever as well as in noninfectious diseases such as Behçet’s disease and Susac’s syndrome [10,11,12]. At the time of writing this report, we could find a single report of Kyrieleis plaques in ARN due to HSV type 1, another one of ARN due to HSV type 2, five reports of ARN due to VZV and a single report of ARN where the causative agent was not specified (Table 2). More specifically, Witmer et al. described a case of unilateral ARN due to HSV type 2. Kyrieleis plaques were observed after treatment which consisted of administration of intravenous acyclovir followed by oral valacyclovir and prednisone in combination with intravitreal foscarnet injections and finally a vitrectomy. Goel et al. presented a case of unilateral ARN due to HSV type 1 with Kyrieleis plaques observed a week after diagnosis. The patient was treated with intravenous acyclovir followed by oral acyclovir and prednisone. Table 2 summarizes published case reports of Kyrieleis arteriolitis in acute retinal necrosis.

To the best of our knowledge, this is the first report of Kyrieleis plaques in ARN secondary to viral encephalitis and the second one that describes the presence of Kyrieleis arteriolitis in ARN due to HSV type 1.

Regardless of its cause, ARN constitutes an ophthalmological emergency that carries significant ocular morbidity. Prompt initiation of treatment is of great importance to prevent the otherwise rapid progression of retinal necrosis and cease the process leading to further deterioration of visual function.

## Figures and Tables

**Table 1:** Patient’s clinical data.

Patient: 52-year-old Caucasian female
Chief complaint and duration: 3-day history of blurred vision, redness, floaters and photophobia in OS
Previous ocular history: Unremarkable
Previous medical history: Herpes simplex virus-1 encephalitis 4 months ago
Best corrected visual acuity: OD: 20/20 OS: 20/33
Anterior segment examination: OD: unremarkable OS: dilated ciliary blood vessels and intense anterior chamber activity
Dilated fundus examination: OD: unremarkable OS: prominent vitritis, coalescing areas of peripheral retinal necrosis from2 o’clock over 6 o’clock to 10 o’clock without involvement of the posterior pole and mild optic disk edema

**Table 2:** Presentation of previous case reports of Kyrieleis arteriolitis in acute retinal necrosis.

Case Report and Year of Publication	Causative Viral Agent	Patient’s Age/Gender	Eye(s) with ARN and KA	Time between Diagnosis of ARN and Observation of KA
Francés-Muñoz et al. [10] (2010)	VZV	76/Female	OU	2 weeks
Witmer et al. [8] (2011)	HSV-2	19/Female	OS	6.5 weeks
Empeslidis et al. [13] (2013)	VZV	56/Male	OS	KA present at diagnosis of ARN
Villena-Irigoyen et al. [14] (2015)	VZV	77/Male	ARN: OU KA: OS	4 weeks
Goel et al. [15] (2016)	HSV-1	55/Male	OS	1 week
Chawla et al. [16] (2017)	VZV	43/Male	ARN: OU KA: OD	Unknown (diagnosis of ARN and description of KA made by different medical teams)
Ning et al. [17] (2018)	VZV	Age not specified/Female	OD	KA present at diagnosis of ARN
Kaza et al. [18] (2020)	Not specified	36/Male	OU	KA present at diagnosis of ARN

ARN: Acute retinal necrosis; KA: Kyrieleis arteriolitis.

## Data Availability

Not applicable.
